# Insight into global research on health literacy and heart diseases: A bibliometric analysis

**DOI:** 10.3389/fcvm.2022.1012531

**Published:** 2022-11-24

**Authors:** Le Duc Huy, Nguyen L. T. Truong, Nhi Y. Hoang, Nhi Thi Hong Nguyen, Thao T. P. Nguyen, Loan T. Dang, Yi-Hsin Elsa Hsu, Chung-Chien Huang, Yao-Mao Chang, Chung-Liang Shih, Elena T. Carbone, Shwu-Huey Yang, Tuyen V. Duong

**Affiliations:** ^1^Health Personnel Training Institute, University of Medicine and Pharmacy, Hue University, Hue, Thua Thien Hue, Vietnam; ^2^School of Medicine, Vietnam National University, Ho Chi Minh City, Vietnam; ^3^Pharmacy Department, Thong Nhat Hospital, Ho Chi Minh City, Vietnam; ^4^School of Nutrition and Health Sciences, Taipei Medical University, Taipei, Taiwan; ^5^School of Health Care Administration, College of Management, Taipei Medical University, Taipei, Taiwan; ^6^Institute for Community Health Research, University of Medicine and Pharmacy, Hue University, Hue, Vietnam; ^7^School of Nursing, National Taipei University of Nursing and Health Sciences, Taipei, Taiwan; ^8^Faculty of Nursing and Midwifery, Hanoi Medical University, Hanoi, Vietnam; ^9^Executive Master Program of Business Administration in Biotechnology, College of Management, Taipei Medical University, Taipei, Taiwan; ^10^International Ph.D. Program in Biotech and Healthcare Management, College of Management, Taipei Medical University, Taipei, Taiwan; ^11^Department of Long-Term Care and School of Gerontology Health Management, College of Nursing, Taipei Medical University, Taipei, Taiwan; ^12^School of Pharmacy, College of Pharmacy, Taipei Medical University, Taipei, Taiwan; ^13^Research Center of Health and Welfare Policy, Taipei Medical University, Taipei, Taiwan; ^14^Ministry of Health and Welfare, Taipei, Taiwan; ^15^Department of Nutrition, University of Massachusetts, Amherst, MA, United States; ^16^Nutrition Research Center, Taipei Medical University Hospital, Taipei, Taiwan; ^17^Research Center of Geriatric Nutrition, Taipei Medical University, Taipei, Taiwan; ^18^International Master/Ph.D. Program in Medicine, College of Medicine, Taipei Medical University, Taipei, Taiwan

**Keywords:** heart diseases, health literacy, bibliometric analysis, instruments, heart failure, ischemic heart diseases

## Abstract

**Background:**

Health literacy (HL) has shown its important role on reducing the burden of heart diseases. However, no study has provided a comprehensive worldwide view of the data regarding HL and heart diseases. The study aimed to provide insight into: (1) the intellectual structure, (2) research trends, and (3) research gaps on HL and heart diseases; and (4) to explore HL scales commonly utilized in heart studies.

**Materials and methods:**

Studies related to HL and heart diseases were retrieved from Web of Science, Scopus, and PubMed. All publications published between 2000 and 2021 were included after conducting keyword searches on “heart diseases” in general or on specific types of heart diseases (e.g., “heart failure”) and “health literacy”. Bibliometric analyses were carried out using the Bibliometrix R package and VOSviewer 1.6.14.

**Findings:**

A total of 388 original research articles and reviews on HL and heart diseases were included in our study. The studies were primarily conducted in the United States and developed countries. A total of 337 studies (86.9%) focused on heart failure (200 studies, 51.5%) and ischemic heart diseases (137 studies, 35.3%). Sixty-two studies (16.0%) focused on other heart diseases (e.g., valvular diseases and rheumatic heart diseases). The number of interventional studies was limited (52 studies, 13.4%) and fluctuated from 2000 to 2021. The most common questionnaires measuring health literacy among patients with heart diseases were the Test of Functional Health Literacy in Adults (TOFHLA), Short Test of Functional Health Literacy in Adults (STOFHLA), and Brief Health Literacy Screen (BHLS). Use of the eHealth Literacy Scale (eHEALS) has become the latest trend among patients with heart diseases.

**Conclusion:**

Health literacy and heart diseases were most often studied in the United States and developed countries. Several HL tools were used; eHEALS has been lately used in this field. These findings suggest the need to conduct more empirical studies on HL and heart diseases in different settings (e.g., developing or poor countries) and with different types of heart diseases (e.g., valvular and rheumatic disorders). Additionally, it is necessary to develop heart disease-specified HL scales for research and practice.

## Introduction

According to the Global Burden of Disease (GBD) Study 2019, cardiac diseases remain the most common cause of mortality globally, with over 9.1 million deaths. Of these, deaths due to ischemic heart disease made up the highest proportion (49.2%) in 2019 (1). Although cardiac risk factor management guidelines emphasize promoting healthy lifestyles, cardiac rehabilitation, and medication use (2, 3), it would be challenging for patients with inadequate health literacy (HL) to adopt and enhance healthy practices (4).

Health literacy indicates the different levels of knowledge and skills to understand, apply and critically analyze health information. Hence, people with adequate HL are able to foster self-management, decision making and many determinants of health (5–7). However, insufficient HL remains a major concern, resulting in restricted access to health services, poor physician-patient communication, unhealthy lifestyle behaviors, and increasing risks of hospital readmission and mortality (8, 9). Therefore, improvement of HL is of utmost importance to enhance patients’ capacity for self-management of their diseases and prevention of prospective cardiac events (10).

As a result of scientometric and big data advancements, bibliometric analysis is gaining more interest to deal with the large amount of data from scientific publications. Bibliometric research focuses on exploring new trends, deciphering potential areas in a particular research discipline, and investigating the contribution of journals, authorship networks, institutes, and countries based on constructing the intellectual structure over the time (11, 12). Bibliometric data are often extracted from large scientific databases including Scopus, Web of Science, PubMed, or Google Scholar. A recent bibliometric study on HL instruments shows that there were 90 health condition-specific HL instruments with a major focus on chronic diseases (13). In addition, to our knowledge, there has been a wealth of systematic reviews and meta-analyses to examine the role of HL in cardiovascular diseases. Yet, no recent bibliometric study has investigated the overall evidence on HL in cardiac disease care and management.

Therefore, we conducted a bibliometric analysis using data from three well-known large databases (Scopus, Web of Science, and PubMed) to systematically investigate all available evidence to create a global view of HL research in patients with heart diseases. We also explored the most common HL instruments used in these patients over time. We expect that our study will support researchers, health policymakers and practitioners in developing strategic interventions to enhance HL and improve the health outcomes of patients with heart diseases.

## Materials and methods

### Data collection and search strategy

We retrieved scientific papers from three databases–Web of Science, Scopus, and PubMed. The search terms related to cardiac diseases were developed based on the International Statistical Classification of Diseases and Related Health Problems 10th Revision (ICD-10) (14). The search terms focused on heart diseases in general and specific types of heart diseases, and HL. We searched and retrieved the relevant data on March 15 2022. Detailed steps of the search process are provided in Table 1.

**TABLE 1:** Search strategies in PubMed, Scopus, and Web of Science.

Database	Search details	Results
Scopus	[TITLE-ABS-KEY (“rheumatic heart disease” OR “rheumatic mitral valve” OR “rheumatic aortic valve” OR “rheumatic tricuspid valve” OR “heart valve disease*” OR “multiple valve disease*” OR “valvular heart disease*” OR “ischemic heart” OR “myocardial infarction” OR “angina pectoris” OR “coronary heart disease*” OR “pulmonary heart disease*” OR “pericarditis” OR “pericardium” OR “endocarditis” OR “myocarditis” OR “cardiomyopathy” OR “heart aneurysm” OR “atherosclerosis” OR “atherosclerotic” OR “arrhythmias” OR “atrial fibrillation” OR “atrial flutter” OR “congenital heart*” OR “coronary artery” OR “heart failure” OR “ventricular failure”) AND TITLE-ABS-KEY (“health literacy” OR “health competenc*”)] AND [Exclude (Publication Years, 2022)] AND [Limit-to (Language, “English”)] AND (Document types, “articles”) OR [Limit-to (Document types, “review articles “)].	592
PubMed	[“rheumatic heart disease”(Title/Abstract) OR “rheumatic mitral valve”(Title/Abstract) OR “rheumatic aortic valve”(Title/Abstract) OR “rheumatic tricuspid valve”(Title/Abstract) OR “heart valve disease*”(Title/Abstract) OR “multiple valve disease*”(Title/Abstract) OR “valvular heart disease*”(Title/Abstract) OR “ischemic heart”(Title/Abstract) OR “myocardial infarction”(Title/Abstract) OR “angina pectoris”(Title/Abstract) OR “coronary heart disease*”(Title/Abstract) OR “pulmonary heart disease*”(Title/Abstract) OR “pericarditis”(Title/Abstract) OR “pericardium”(Title/Abstract) OR “endocarditis”(Title/Abstract) OR “myocarditis”(Title/Abstract) OR “cardiomyopathy”(Title/Abstract) OR “heart aneurysm”(Title/Abstract) OR “atherosclerosis”(Title/Abstract) OR “atherosclerotic”(Title/Abstract) OR “arrhythmias”(Title/Abstract) OR “atrial fibrillation”(Title/Abstract) OR “atrial flutter”(Title/Abstract) OR “congenital heart*”(Title/Abstract) OR “coronary artery”(Title/Abstract) OR “heart failure”(Title/Abstract) OR “ventricular failure”(Title/Abstract) OR “Heart Diseas*”(MeSH Terms) OR “Heart disorde*”(MeSH Terms)] AND [“Health Literacy”(MeSH Terms) OR “Health Literacy”(Title/Abstract) OR “health competenc*”(Title/Abstract)].	460
Web of Science	TS = [(“heart disease*” OR “heart disorder*” OR “rheumatic heart disease*” OR “Heart Valve Diseases” OR “Ischemic heart diseases” OR “myocardial infarction” OR “pulmonary heart diseases” OR “pericarditis” OR “pericardium” OR “myocarditis” OR “cardiomyopathy” OR “arrhythmias” OR “cardiac arrhythmias” OR “Atrial fibrillation*” OR “Heart failure*” OR “Atherosclerotic*” OR “Atherosclerosis*” OR “Coronary artery*” OR “congenital heart” OR “heart defect*”) AND (“health literac*” OR “health competenc*”)] AND [Exclude (Publication Years, 2022)] AND [Limit-to (Language, “English”)] AND Limit-to (Document types, “articles”) OR (Limit-to (Document types, “review articles”)] AND [Limit-to (Web of Science Index, “Science Citation Index Expanded (SCI-EXPANDED) or Social Sciences Citation Index (SSCI)].	524

We included original research articles and reviews written in English and published between 2000 and 2021 in the analysis. Grey literature, conference proceedings, books/book review, letters, case reports, congressional papers, editorials, comments, and news were excluded.

### Data screening

**FIGURE 1:**
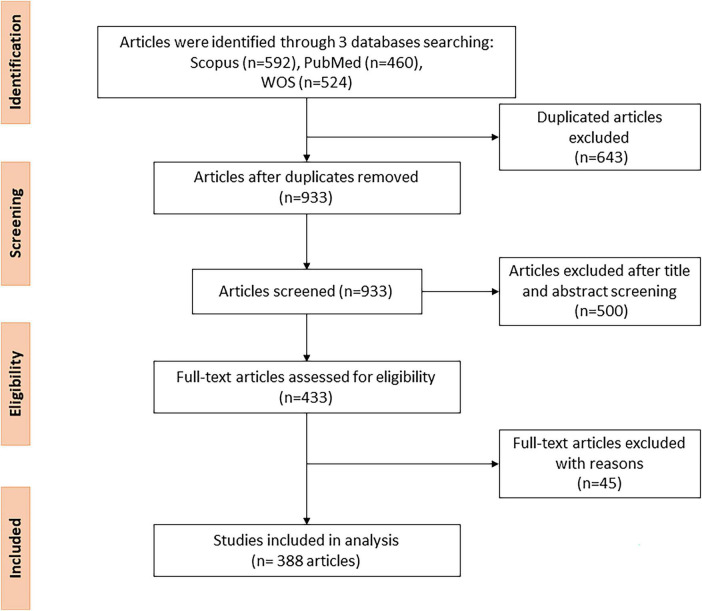
The PRISMA flow diagram.

We used the Bibliometrix R package to combine data from various sources and remove duplication (15). Researchers screened article titles and abstracts to identify relevant papers and extract bibliometric parameters. During the extraction process, we focused on the following key bibliometric parameters: year of publication, citation, country, journal, study design, types of heart disease, and HL questionnaires. To identify the HL instruments, the researchers manually screened the full text in all the studies and created a comprehensive list of HL questionnaires. Then, we combined the uncommon HL instruments into groups with similar applications. After screening, 388 papers were included in the final analysis. We employed PRISMA for the extraction flow (16). The data extraction process is presented in Figure 1.

### Analysis

We used the Bibliometrix R package (15) and Microsoft Excel (version 2013) to analyze and visualize the bibliometric parameters. The VOS Viewer (version 1.6.14) was utilized to carry out the cluster analysis, co-authorship analysis, and to establish the network maps (nodes and links) for countries and authors (17).

## Results

### General characteristics

**FIGURE 2:**
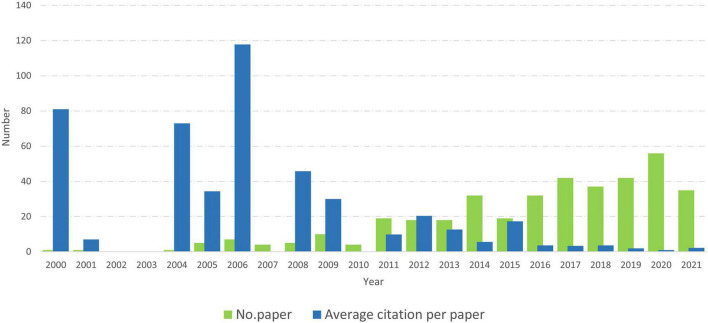
The growth rate of publications by year.

Figure 2 shows the growth rate of publications by year. In general, the number of publications has increased, while the average citations decreased, respectively by year. Specifically, the number of publications increased gradually over the study period and significantly grew after 2010. In particular, the number of publications peaked at 56 publications in 2020. Notably, the number of publications dropped significantly in 2021. On the other hand, the average citation per paper ranked the highest in 2006, with 118 citations.

**FIGURE 3:**
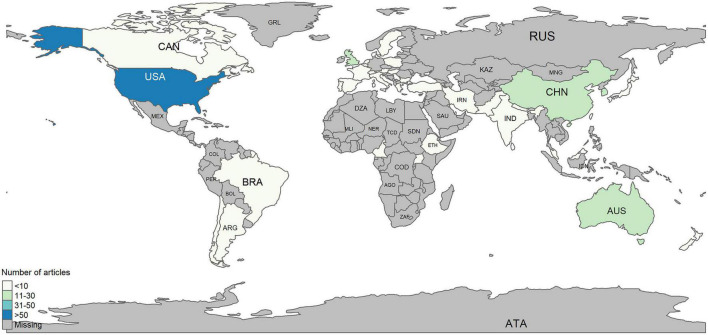
Global maps of publication by countries.

Figure 3 illustrates the number of publications by country. Overall, heart disease-related HL studies were conducted in 45 nations. The United States contributed the most publications, with 202 articles, followed by Australia (25 articles), and China (14 articles). Canada, Brazil, Argentina, Iran, India, Egypt, and others have less than ten articles, as indicated by the global maps.

Co-authorship analyses were conducted to investigate the cooperation network between authors and nations, wherein publications with a stronger overall link strength between authors and countries indicate a tighter relationship (18).

**FIGURE 4:**
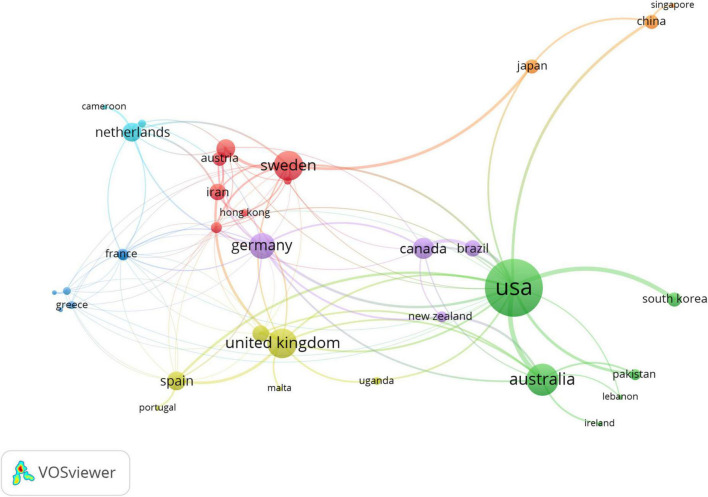
The country co-authorship network of heart diseases related publications.

As shown in Figure 4, the United States has the highest network of co-authorship in the heart disease-related research among countries, followed by Australia, the United Kingdom, Sweden, and Germany. Other representatives of Europe (e.g., Netherlands, Austria, and Spain), North America (e.g., Canada), and Asia (e.g., Japan, South Korea, and China) have often collaborated with the leading co-authorship countries in this field.

**FIGURE 5:**
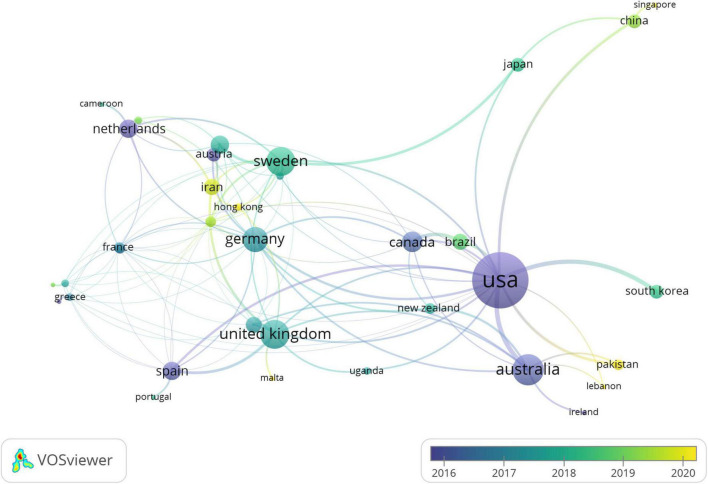
The country co-authorship network of heart diseases related publications over the time.

Figure 5 illustrates the co-authorship network by countries who conducted heart diseases-related studies over time. The United States, Australia, Netherlands, Canada, and Spain (in purple) have a long history of heart disease research. Conversely, Iran, Hong Kong, Pakistan, and China (in yellow) joined this research field in the previous few years.

As shown in Table 2, the Patient Education and Counseling journal has published the highest number of papers, with 20 articles accounting for 5.15% of the total journals publishing about HL and heart diseases. In addition, articles in the Journal of General Internal Medicine had the highest number of citations per paper (53.58) and total citations (643).

**TABLE 2:** Top 10 journals published health literacy articles related to heart diseases.

Journal	No. papers	Percentage of total articles	Total citation	No. citations per article
Patient Education and Counseling	20	5.15	233	11.65
Journal of General Internal Medicine	12	3.09	643	53.58
The Journal of Cardiovascular Nursing	11	2.84	0	0.00
Journal of Cardiac Failure	9	2.32	83	9.22
PLoS One	8	2.06	38	4.75
BMJ Open	7	1.80	31	4.43
European Journal of Preventive Cardiology	7	1.80	71	10.14
International Journal of Environmental Research and Public Health	7	1.80	0	0.00
Mayo Clinic Proceedings	6	1.55	135	22.50
BMC Health Services Research	5	1.29	238	47.60

**FIGURE 6:**
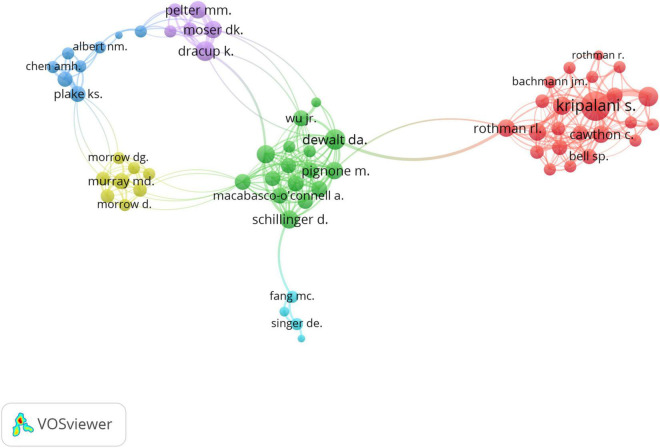
The distribution of co-authorship network.

Figure 6 illustrates the network map of co-authorship. There were six different color clusters of close cooperation (with at least three papers). The thickness of the lines indicates the strength of the relationship. For example, Kripalani S. cooperated closely with Cawthon C., Rothman RL., Rothman R., Bachmann IM., and Bell SP (Red cluster). In addition to being in close contact with co-authors in the green cluster, DeWalt DA. frequently collaborated with Rothman R., Dracup K., etc. Our findings indicate that the two most prolific group of authors include Kripalani S. and colleagues (Green cluster in Figure 6) and DeWalt D.A. and colleagues (Red cluster in Figure 6). The first group mainly focuses on medical adherence (19–21), medication errors (22, 23) and health outcomes regarding mortality (24, 25) and readmission risk (26). On the other hand, DeWalt’s group mainly conducted research on the relationship between the HL and heart failure (27–29). Apart from the well-established research groups, there were emerging research teams (e.g., Plake Kimberly S., Albert Nancy M., Chen Aleda M.H.) who actively investigated the role of digital HL in cardiac outcomes (30–32).

**FIGURE 7:**
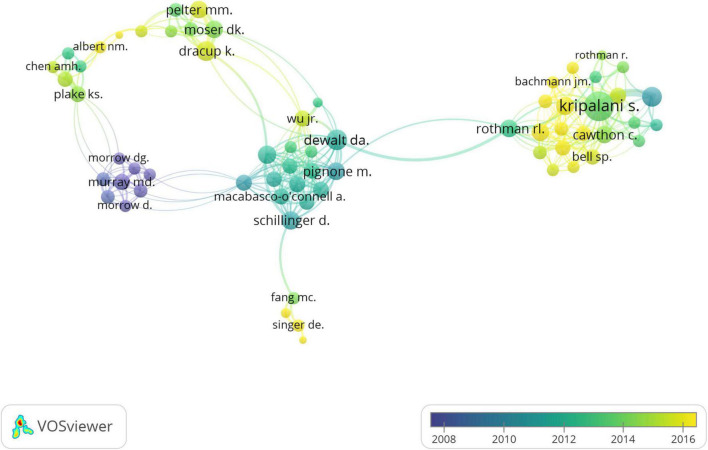
Distribution of co-authorship network over the time.

Considering the co-authorship network between 2008 and 2016 (Figure 7), Morrow DG., Murray MD., and Morrow D. (in purple) have collaborated since 2008 in the HL research related to heart diseases. Moreover, Kripalani S. has joined this research field in the past few years and actively expanded his collaboration network with other authors.

### Classification of health literacy research related to heart diseases by study design

**FIGURE 8:**
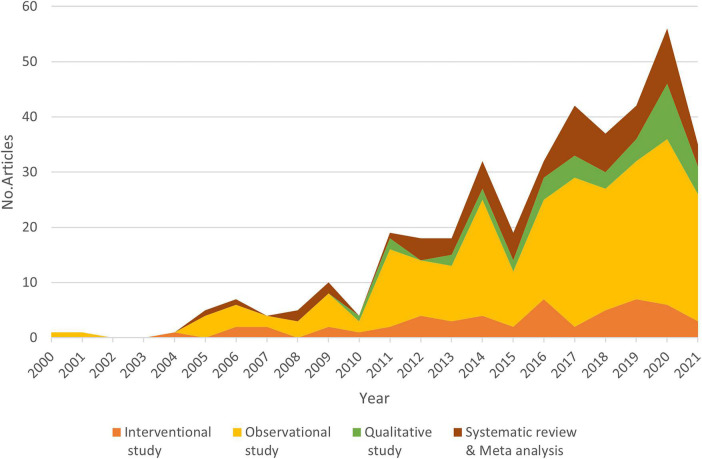
The distribution of papers classified by study design over the time.

Figure 8 indicates that observational studies were a dominant study design between 2004 to 2021. The number of papers on qualitative studies, systematic reviews, and meta-analyses also increased steadily after 2010. Meanwhile, the number of interventional studies varied during this period of time and reached the peak in 2016 and 2019 with 7 to 8 articles, respectively.

Table 3 shows that among 20 countries, the United States had the highest number of papers (*n* = 202) and the number of papers utilizing an observational study design was the highest (125/202, 61.9%). Australia ranked second regarding the number of papers (*n* = 25). The number of papers from other countries; namely, Germany, the United Kingdom, South Korea, and China, ranged from 10 to 14. Meanwhile, Austria and New Zealand had the smallest number of papers (three papers for each).

**TABLE 3:** The number of papers with different study designs in top 20 productive countries.

Country	Interventional study	Observational study	Qualitative study	Reviews	Total
The United States	34	125	12	31	202
Australia	2	9	7	7	25
China	0	14	0	0	14
South Korea	1	10	0	1	12
United Kingdom	1	4	2	4	11
Germany	1	5	0	4	10
Sweden	1	3	5	0	9
Canada	0	1	4	3	8
Iran	2	5	0	1	8
Spain	1	6	0	0	7
Brazil	0	6	0	0	6
Denmark	3	1	2	0	6
Netherlands	0	5	1	0	6
Poland	0	5	0	1	6
Japan	0	5	0	0	5
Taiwan	1	3	1	0	5
France	0	3	0	1	4
India	0	3	1	0	4
Austria	1	2	0	0	3
New Zealand	1	1	1	0	3

### Classification of health literacy research by cardiac disease categories

Table 4 presents the top six heart diseases investigated in the top 20 countries, including atrial fibrillation, cardiac valve disorders, congenital cardiac malformations, chronic rheumatic heart diseases, ischemic heart diseases, and heart failure. Among those, most were heart failure (189/388 articles, 48.7%), and the United States conducted the majority of studies (202/388, 52.0%).

**TABLE 4:** Number of papers regarding six most common heart diseases in top 20 productive countries.

Country	Heart failure	Atrial fibrillation and flutter	Ischemic heart disorders	Cardiac valve disorders	Chronic rheumatic heart diseases	Congenital malformations of heart components	Other	Total
The United States	118	17	67	1	1	3	2	202
Australia	10	6	7	0	1	2	0	25
China	6	0	8	0	0	0	0	14
South Korea	9	1	1	0	0	0	0	12
The UK	3	0	8	0	1	0	0	11
Germany	3	1	7	1	0	0	1	10
Sweden	4	0	2	0	0	2	1	9
Canada	4	1	3	0	0	0	0	8
Iran	6	1	1	0	0	0	0	8
Spain	5	1	1	1	0	0	0	7
Brazil	3	2	2	1	0	0	0	6
Denmark	2	0	5	1	0	0	0	6
Netherlands	2	1	4	0	0	0	0	6
Poland	3	2	0	1	0	0	0	6
Japan	5	0	0	0	0	0	0	5
Taiwan	3	0	1	0	1	1	0	5
France	2	1	0	0	0	1	0	4
India	1	1	2	1	1	0	0	4
Austria	0	1	2	0	0	0	0	3
New Zealand	0	0	2	0	1	0	0	3
Total	189	36	123	7	6	9	4	

Supplementary Figure 1 presents the distribution of studies on different types of heart diseases. Heart failure and ischemic heart diseases accounted for the most significant number of publications over the study period. After 2015, HL research related to atrial fibrillation and flutter witnessed a considerable growth in publications, and the number of studies about congenital malformations or cardiac valvular diseases increased slightly.

Table 5 shows citation analysis across heart disease types. Ischemic heart diseases had the highest average citation per paper (10.52), followed by heart failure and atrial fibrillation diseases. Although ischemic heart diseases account for the highest mortality and morbidity burden (2,421 patients and 118 deaths per 100,000 inhabitants), heart failure is the focus of most publications related to HL globally (with over 50% publications).

**TABLE 5:** Number of papers regarding six most popular heart diseases among different study design.

Heart diseases (ICD code)	Prevalence (Deaths)^a^	Indicators	Interventional study	Observational study	Qualitative study	Review study	Overall
Chronic rheumatic heart diseases (I05–I09)	513.68 (3.85)	Count	0	3	3	1	7
		No. citation per paper	0	6.67	0	0	2.86
		Total citation	0	20	0	0	20
Congenital malformations of cardiac components (Q20–Q24)	183.45 (3.23)	Count	0	5	4	1	10
		No. citation per paper	0	1.4	2.25	0	1.6
		Total citation	0	7	9	0	16
Ischemic heart diseases (I20–I25)	2421.02 (117.95)	Count	21	84	13	19	137
		No. citation per paper	4	13.48	6.46	7.42	10.52
		Total citation	84	1132	84	141	1441
Atrial fibrillation and flutter (I48)	743.47 (4.38)	Count	3	26	2	7	38
		No. citation per paper	0.67	10.65	3.5	0.43	7.61
		Total citation	2	277	7	3	289
Heart failure (I50)	233.77 (15.16)	Count	31	113	18	38	200
		No. citation per paper	19.1	5.27	2.78	19.84	9.96
		Total citation	592	595	50	754	1991
Cardiac valve disorders (I35, T82)	399.5 (2.25)	Count	1	6	0	0	7
		No. citation per paper	0	29.83	0	0	25.57
		Total citation	0	179	0	0	179

^a^Data were extracted from the Global burden databases 2019.

### Health literacy instruments

**FIGURE 9:**
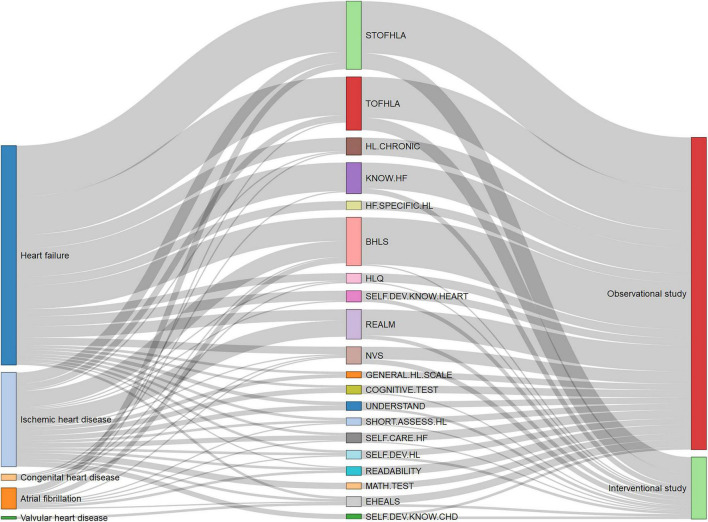
Distribution of health literacy (HL) instrument usage across heart diseases and type of study designs. BHLS, Brief Health Literacy Screen; COGNITIVE.TEST, Cognitive tests; EHEALS, eHealth literacy scale; GENERAL.HL.SCALE, General health literacy scale; HF.SPECIFIC.HL, Heart Failure-Specific Health Literacy Scale; HL.CHRONIC, Health Literacy in Patients with Chronic Diseases; HLQ, Health literacy questionnaire; KNOW.HF, Questionnaire about Heart Failure Patients Knowledge of Disease; MATH.TEST, Numeracy tests; NVS, Newest Vital Sign; READABILITY, Questionnaires about the readability of patient education materials; REALM, Rapid Estimate of Adult Literacy in Medicine; SELF.CARE.HF, Questionnaire about self-care and heart failure; SELF.DEV.HL, Self-developed questionnaires about health literacy; SELF.DEV.KNOW.CHD, Self-developed questionnaires about knowledge of coronary heart disease; SELF.DEV.KNOW.HEART, Self-developed questionnaires about knowledge of heart diseases; SHORT.ASSESS.HL, Short Assessment of Health Literacy; STOFHLA, Short Test of Functional Health Literacy in Adults; TOFHLA, Test of Functional Health Literacy in Adults; UNDERSTAND, Questionnaire about understandability.

Figure 9 shows the distribution of HL questionnaire usage for different heart diseases and types of study designs. The results show that STOFHLA and TOFHLA were the two most popular questionnaires administered to measure HL among patients with varying heart conditions. Other popular questionnaires for HL measurement among cardiac patients included BHLS, REALM, and NVS. Apart from these widely validated questionnaires, many studies used self-developed questionnaires to study knowledge of a specific disease such as coronary heart disease (CHD). Only health literacy of patients with heart failure were assessed using validated instruments such as a questionnaire about self-care and heart failure and heart failure-specific health literacy scale. STOFHLA and TOFHLA were widely utilized in observation and intervention studies. BHLS and REALM were more likely to be used in observational studies rather than the interventional ones.

**FIGURE 10:**
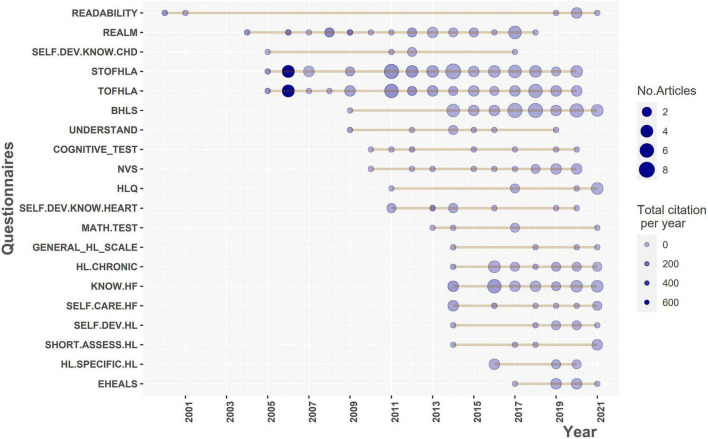
Top 20 questionnaires of health literacy (HL) used over the time. BHLS, Brief Health Literacy Screen; COGNITIVE.TEST, Cognitive tests; EHEALS, eHealth literacy scale; GENERAL.HL.SCALE, General health literacy scale; HF.SPECIFIC.HL, Heart Failure-Specific Health Literacy Scale; HL.CHRONIC, Health Literacy in Patients with Chronic Diseases; HLQ, Health literacy questionnaire; KNOW.HF, Questionnaire about Heart Failure Patients Knowledge of Disease; MATH.TEST, Numeracy tests; NVS, Newest Vital Sign; READABILITY, Questionnaires about the readability of patient education materials; REALM, Rapid Estimate of Adult Literacy in Medicine; SELF.CARE.HF, Questionnaire about self-care and heart failure; SELF.DEV.HL, Self-developed questionnaires about health literacy; SELF.DEV.KNOW.CHD, Self-developed questionnaires about knowledge of coronary heart disease; SELF.DEV.KNOW.HEART, Self-developed questionnaires about knowledge of heart diseases; SHORT.ASSESS.HL, Short Assessment of Health Literacy; STOFHLA, Short Test of Functional Health Literacy in Adults; TOFHLA, Test of Functional Health Literacy in Adults; UNDERSTAND, Questionnaire about understandability.

Figure 10 indicates the frequency of questionnaire utilization and their citations over time. Before 2011, most studies on heart disease and HL utilized general HL questionnaires. A typical tool was the readability test of patient education materials, which has been used since the very beginning of the domain and continues to be applied in recent years. The other questionnaires, notably TOFHLA, STOFHLA, and REALM, have a long history and widespread use in HL research. Although general HL measures continued to dominate after 2011, the number of studies utilizing questions focused on cardiac illness increased significantly, primarily related to heart failure. The short versions of the HL scale also increased in recent years (e.g., BHLS, STOFHLA, short assessment HL, NVS). After 2017, there was notable growth in the number of studies focusing on digital HL, with increased usage of the EHEALS tool. The full list of questionnaires is available in Supplementary Figure 2.

## Discussion

To our knowledge, this is the first bibliometric analysis of the evidence of HL related to cardiac diseases, which comprehensively includes systematic reviews, meta-analysis, interventional studies, and observational studies in the three largest databases (Web of Science, Scopus, and PubMed).

Our results show that the number of publications increased gradually over the study period and experienced significant growth after 2010. However, in 2021, the number of studies dropped significantly. A possible reason could be the influence of COVID-19 on research activities (33). The restriction on gatherings, workplace closures and internal movement restrictions might have created various challenges in conducting research projects. Observational studies were the dominant study design during the study period. In the latter phase of the study period, the number of interventional studies, systematic reviews, and meta-analyses witnessed an upward trend. Since the HL field has become more mature and gained more attention from different researchers and societies, the types of study designs have become more diverse.

### Authorship and country collaborations

The role of HL in cardiac disease has gained attention from global researchers (9). HL studies related to heart diseases have been investigated in 45 countries, with most studies carried out in developed countries. Of these, the United States made the largest contribution to this field, accounting for over 50% of the studies. Previous bibliometric research related to cardiac diseases also showed similar results, with the United States emerging as the most prolific country in research quantity (34, 35).

According to the results of the country’s collaboration network, we found that besides traditional collaborations among well-developed western countries (e.g., The United States, Australia, Canada, Netherlands), increasingly strong research networks among developing countries have been established in recent years, particular in Asia. This might be due to the fact that most Asian countries are in the second stage of a rapidly increasing cardiovascular diseases (CVD) epidemic (36). A recent study showed that the number of CVD deaths in Asia doubled (from 5.6 million to 10.8 million), and the proportion of CVD deaths in total deaths increased (from 23% to 35%) between 1990 and 2019 (36). In addition, there were increasing lifestyle-related risk factors (e.g., tobacco, hypertension, and obesity) (37).

In terms of authorship networks, we found that the largest research groups usually include the leading and most prolific authors in the field. Our findings support the idea that building up a large and strong co-authorship network is crucial for increasing the productivity of publications in the field (35).

### Types of heart disease

In the present study, heart failure and ischemic heart diseases gained most researchers’ attention. These two diseases accounted for the highest prevalence and mortality rates in high-income countries where made the largest contribution to the structure of HL research related to heart diseases, meanwhile, the prevalence and mortality of rheumatic diseases and congenital heart diseases were much more popular in low-to-middle income countries (38). In addition, previous meta-analyses have shown that HL could influence self-care behaviors and other health outcomes among patients suffering from heart failure (39). Although the literature on chronic rheumatic heart disease and cardiac valve disorders was very limited, these kinds of diseases are creating the largest heart related health burdens on society, even larger than heart failure in low-to-middle income countries. The lack of evidence on the role of HL in heart diseases might be a barrier to improving and initiating new strategies for the prevention and advancement of treatments for cardiac diseases. We suggest that further study is necessary to clarify the role of HL in patients with these diseases.

### Health literacy questionnaires

Our findings indicate that TOFHLA, STOFHLA, and BHLS were the most common instruments used for measuring HL among patients with heart diseases. The TOFHLA was developed by Ruth M. Parker et al. in 1995, consisting of 67 questions (50-item reading comprehension and 17-item numerical ability test) (40). The questionnaire was frequently used to evaluate patients’ ability to read and understand information they often encounter in health care settings (40). In 1999, David W. Baker developed an abbreviated version of TOFHLA, including 40 questions, which reduced completion time by 10 min compared to TOFHLA (41). The short-form version (STOFHLA) has demonstrated comparable reliability and validity to the previous version but is more practical due to its shorter administration time and fewer complicated questions (41). The BHLS questionnaire was developed by Chew et al. to screen patients with inadequate HL (42). Since the instrument only includes three items, it can increase the feasibility of use in crowded clinical settings and quickly identify the patients who need special communication methods (42). Despite being developed in 2004, the number of research studies utilizing the BHLS increased significantly after 2015.

Interestingly, an increasing number of recent studies have been using HL questionnaires focused on heart diseases. A typical example is heart failure specific HL developed by Matsuoka et al. (43); this questionnaire includes 12 items measuring comprehensive HL initiated by World Health Organization (5, 44). Apart from the functional HL domain mainly focused on readability, the instrument added more questions to measure the ability to access information (communicative HL) and critically evaluate that information (critical HL) (44).

After 2017, a new trend in evaluation focused on the use of the eHealth Literacy Scale (EHEALS) among patients with heart diseases. The questionnaire was developed by Norman et al. to measure patients’ knowledge and perceived skills related to researching, criticizing, and adopting electronic health information related to health problems (45). Recent bibliometric research demonstrates that eHealth is becoming a trending issue, with the number of studies rapidly growing over the past 5 years, particularly during the COVID-19 epidemic (46). Therefore, further studies investigating the role of eHealth Literacy in specific heart diseases would be essential.

Our study had some limitations. First, we only included studies written in English; hence, we may have missed articles published in other languages. Second, we did not examine the quality of methodological studies. Since there are various types of research designs with different subtypes, it would be challenging to identify a standard and universal method for evaluating the quality of various study designs. However, further analysis of quality would be useful in a future study. Our research did not include all databases, such as Embase and clinical trials. It is therefore possible that we missed some studies published in these databases.

Comparison with prior work, our study has some outstanding strengths. We included the three most popular published databases: PubMed, Web of Science, and Scopus. Therefore, our results are more inclusive as compared to prior studies, which focused on only one of the aforementioned databases (34, 35, 46). To our knowledge, this is the first study to provide a comprehensive picture of the HL tools used in observational and interventional studies related to heart diseases. Knowing which instruments are most often used in HL would be useful to find and select the appropriate tool for research related to cardiac diseases. In addition, researchers might update the latest research instruments with more specificity for different purposes or diseases based on the historical and frequent usage of HL tools.

## Conclusion

Based on our findings, we conclude that apart from two common heart diseases (heart failure and heart ischemic diseases), it is necessary to carry out more research on other types of heart diseases (including valvular disorders and rheumatic disorders). The number of interventions studies is limited and fluctuated over the study period. More HL intervention research would be beneficial to provide initiatives in improving outcomes of heart disease treatment and management. Finally, development of HL scales for different types of heart diseases would also be essential to further research in this area.

## Data availability statement

The original contributions presented in the study are included in the article/Supplementary material, further inquiries can be directed to the corresponding authors.

## Ethics statement

Ethical review and approval was not required for this study in accordance with the local legislation and institutional requirements. Written informed consent was not required for this study in accordance with the local legislation and institutional requirements.

## Author contributions

LH, TD, and S-HY designed the research. LH, TD, S-HY, LD, NT, and NH conducted the literature review. LH, NT, NH, NN, TN, and LD collected the primary data. LH, TD, S-HY, Y-HH, and EC analyzed and visualized the data. NT, NH, NN, Y-MC, Y-HH, C-CH, and C-LS screened out the irregular literature. LH, TD, NT, NH, NN, TN, LD, and S-HY wrote the original draft. LH, TD, C-CH, Y-MC, C-LS, Y-HH, and EC reviewed and revised the manuscript. All authors contributed to the article and approved the submitted version.
