# Supplementary material for: Insight into global research on health literacy and heart diseases: A bibliometric analysis
Source: Front Cardiovasc Med. 2022 Nov 24;9:1012531. doi: 10.3389/fcvm.2022.1012531 (PMC9729531; doi:10.3389/fcvm.2022.1012531)
Supplement: Supplementary file 1 [file Presentation_1.PDF]

Supplementary Material

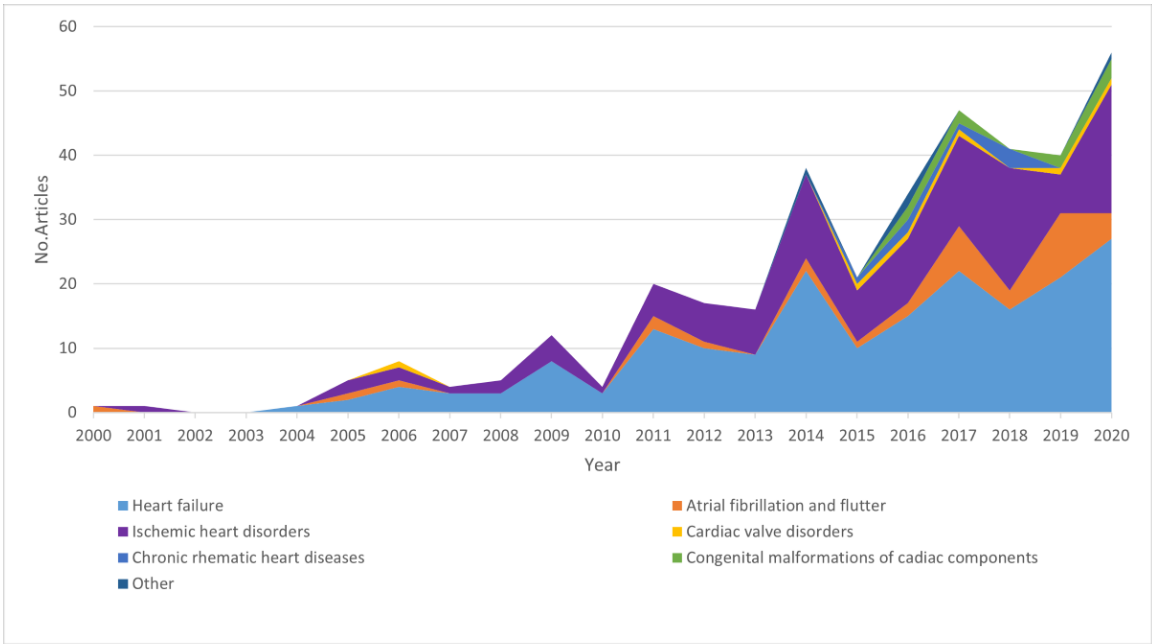

Supplementary Figure 1. Number of studies by type of heart diseases over the study period.

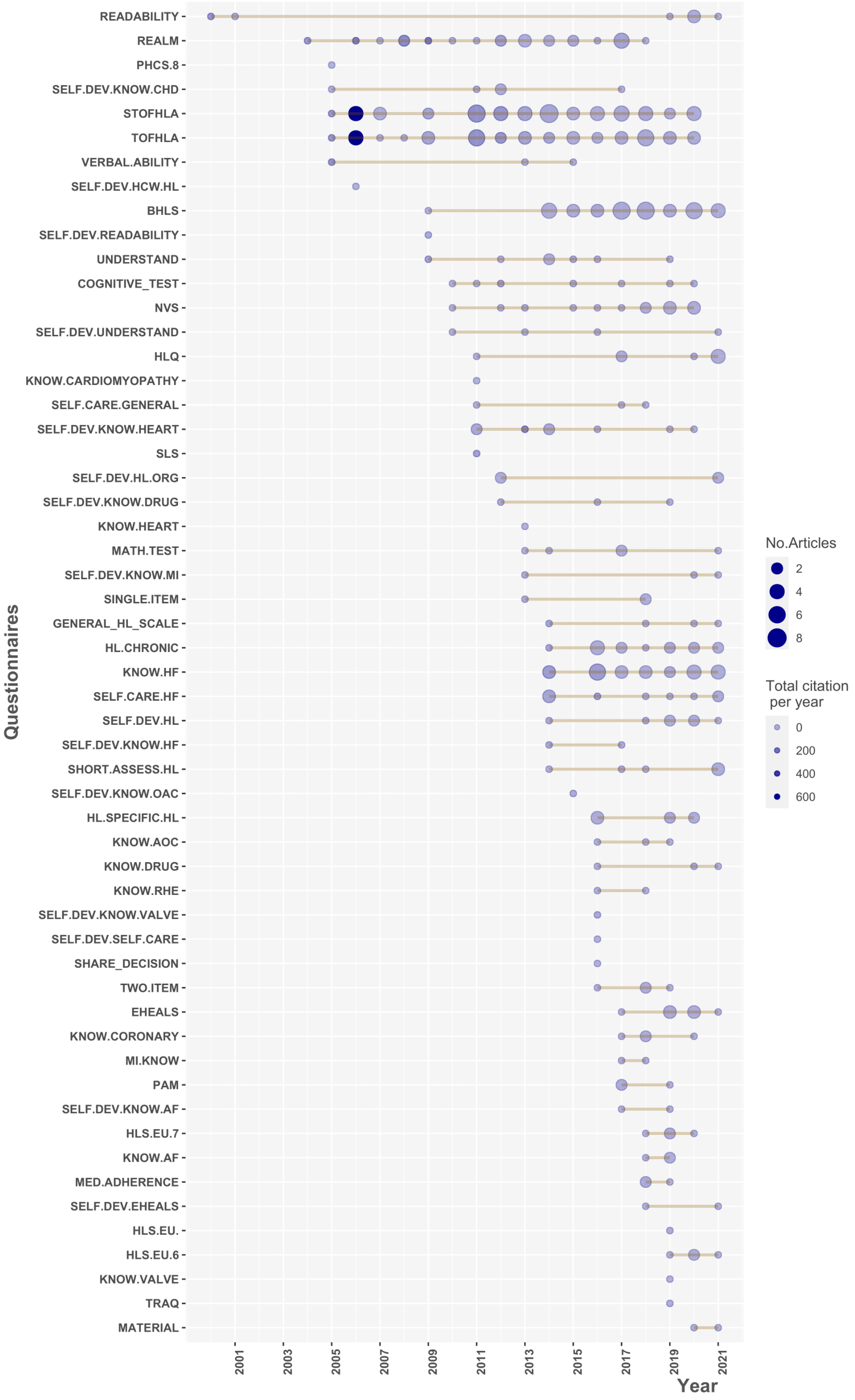

Supplementary Figure 2. List of questionnaires related to health literacy

Abbreviations of Health Literacy Questionnaires

1. READABILITY: Questionnaires about readability
2. REALM: Rapid Estimate of Adult Literacy in Medicine
3. PHCS.8: 8-Item Perceived Health Competence Scale
4. SELF.DEV.KNOW.CHD: Self-developed Questionnaires about Knowledge of Coronary Heart Disease)
5. STOFHLA: Short Test of Functional Health Literacy in Adults
6. TOFHLA: Test of Functional Health Literacy in Adults
7. VERBAL.ABILITY: Questionnaires about Verbal Communication of Patients
8. SELF.DEV.HCW.HL: Self-developed Questionnaires about Health Literacy of Health Care Workers
9. BHLS: Brief Health Literacy Screen
10. SELF.DEV.READABILITY: Self-developed Questionnaires about Readability
11. UNDERSTAND: Questionnaire about Understandability
12. COGNITIVE.TEST: Cognitive tests
13. NVS: Newest Vital Sign
14. HLQ: Health Literacy Questionnaire
15. KNOW.CARDIOMYOPATHY: Questionnaires about Knowledge of Cardiomyopathy
16. SELF.CARE.GENERAL: Questionnaires about Self-care Behaviors
17. SELF.DEV.KNOW.HEART: Self-developed questionnaires about Knowledge of Heart Diseases
18. SLS: Short Literacy Survey
19. SELF.DEV.HL.ORG: Self-developed Questionnaires about Health Literacy Organizations
20. SELF.DEV.KNOW.DRUG: Self-developed Questionnaires about Knowledge of Drugs
30. SELF.DEV.KNOW.HF: Self-developed Questionnaires about Knowledge of Heart Failure
31. SHORT.ASSESS.HL: Short Assessment of Health Literacy
32. SELF.DEV.KNOW.OAC: Self-developed Questionnaires about Knowledge of Oral Anticoagulant
33. HF.SPECIFIC.HL: Heart Failure-Specific Health Literacy Scale
34. KNOW.AOC: Questionnaires about Knowledge of Oral Anticoagulant
35. KNOW.DRUG: Questionnaires about Knowledge of Drugs
36. KNOW.RHE: Questionnaires about Knowledge of Rheumatic Heart Disease
37. SELF.DEV.KNOW.VALVE: Self-developed Questionnaires about Knowledge of Valvular Heart Diseases
38. SELF.DEV.SELF.CARE: Self-developed Questionnaires about Self-care Behaviors
39. SHARE_DECISION: COMRADE survey—combined outcome measure for Risk Communication and Treatment Decision Making Effectiveness
40. TWO.ITEM: Two-item Perceived Health Competence Scale
41. EHEALS: eHealth Literacy Scale
42. KNOW.CORONARY: Questionnaires about Knowledge of Coronary Heart Diseases
43. MI.KNOW: Questionnaires about Knowledge of Myocardial Infarction
44. PAM: Patient Activation Measure questionnaire
45. SELF.DEV.KNOW.AF: Self-developed Questionnaires about Atrial Fibrillation
46. HLS.EU.7: 47-Item European Health Literacy Survey
47. KNOW.AF: Questionnaires about Knowledge of Atrial Fibrillation

21. KNOW.HEART: Questionnaires about Knowledge of Heart Diseases
22. MATH.TEST: Numeracy Tests
23. SELF.DEV.KNOW.MI: Self-developed Questionnaires about Knowledge of Myocardial Infarction
24. SINGLE.ITEM: Single-Item Health Literacy Scale
25. GENERAL_HL_SCALE: General Health Literacy Scale
26. HL.CHRONIC: Health Literacy in Patients with Chronic Diseases
27. KNOW.HF: Questionnaire about Heart Failure Patients' Knowledge of Disease
28. SELF.CARE.HF: Questionnaire about Self-care and Heart Failure
29. SELF.DEV.HL: Self-developed Questionnaires about Health Literacy
48. MED.ADHERENCE: Questionnaires about Medication Adherence
49. SELF.DEV.EHEALS: Self-developed Questionnaires about eHealth Literacy
50. HLS.EU: European Health Literacy Survey
51. HLS.EU.6: 16-Item European Health Literacy Survey
52. KNOW.VALVE: Questionnaires about Knowledge of Valvular Heart Diseases
53. TRAQ: Transition Readiness Assessment Questionnaire
54. MATERIAL: Patient Education Materials Assessment Tool for Print Materials)
